# Capacity and cost benefits of subcutaneous versus intravenous pertuzumab/trastuzumab: The EASE-SC study

**DOI:** 10.1016/j.breast.2025.104573

**Published:** 2025-09-10

**Authors:** Michiel Zietse, Jacky Hu, Esther R. van Staveren, Leontine E.A.M.M. Spierings, Agnes Jager, Birgit C.P. Koch, Ron H.J. Mathijssen, Roelof W.F. van Leeuwen, Frederick W. Thielen

**Affiliations:** a Department of Hospital Pharmacy, Erasmus University Medical Center, Rotterdam, Netherlands; b Department of Internal Medicine, Alrijne Hospital, Leiderdorp, Netherlands; c Department of Medical Oncology, Erasmus University Medical Centre, Rotterdam, Netherlands; d Department of Health Technology Assessment, Erasmus School of Health Policy and Management, Erasmus University Rotterdam, Rotterdam, Netherlands; e Erasmus Centre for Health Economics Rotterdam (EsCHER), Erasmus University Rotterdam, Rotterdam, Netherlands

**Keywords:** HER2-Positive breast cancer, Pertuzumab and trastuzumab, Subcutaneous administration, Intravenous administration, Healthcare resource utilization, Cost analysis, Microcosting

## Abstract

**Objectives:**

Subcutaneous administration of pertuzumab and trastuzumab offers a faster alternative to intravenous infusion for patients with HER2-positive breast cancer. However, real-world data on its impact on costs and capacity remain limited. Therefore, this study aimed to compare healthcare resource utilization and costs associated with subcutaneous versus intravenous administration of pertuzumab and trastuzumab from a societal perspective.

**Methods:**

This study was conducted at two Dutch hospitals. Observational data were collected on drug preparation, administration times, and resource use for both formulations. Patient questionnaires assessed societal costs, including travel expenses and productivity losses. Costs were calculated for patient chair time, healthcare professional time, disposables, societal expenses, and drug costs. A nationwide impact analysis estimated potential capacity and productivity gains from switching from intravenous to subcutaneous administration across the Netherlands.

**Results:**

Subcutaneous administration reduced patient chair time compared to intravenous administration, by an average of 106 min (85.5%) for maintenance doses (from 124.3 to 18.1 min) and 287 min (96.0%) for loading doses (from 299.0 to 12.0 min). Active healthcare professional time decreased by 17 min (54.1%) for maintenance doses and 25 min (66.7%) for loading doses. Drug administration costs (excluding drug costs) were lower subcutaneous administration saved approximately €172 per maintenance dose and €403 per loading dose. Nationwide adoption could create capacity for around 22,000 additional treatments annually and save 4.0 full-time equivalent healthcare professionals.

**Conclusion:**

Switching from intravenous to subcutaneous pertuzumab/trastuzumab administration substantially reduces healthcare resource use and may offer cost savings, supporting more efficient delivery of HER2-targeted therapies.

## Highlights

• Subcutaneous pertuzumab/trastuzumab reduces chair time by over 100 min.
• Healthcare professional time halved with subcutaneous administration per dose.
• Subcutaneous administration of pertuzumab/trastuzumab leads to potential societal cost savings.

## 1 Introduction

Human epidermal growth factor receptor 2 (HER2) is overexpressed in approximately 20% of breast cancer patients, which is associated with poor prognosis and reduced survival rates. [1] The combination of intravenous pertuzumab, trastuzumab, and chemotherapy has demonstrated significant clinical benefits for patients with HER2-positive breast cancer, both in early-stage and metastatic disease. [[2], [3], [4]]

Conventionally, pertuzumab and trastuzumab (PT) are administered intravenously, requiring up to 2.5 h of chair time per patient. [5,6] However, the administration of intravenous anticancer drugs places a considerable burden on oncology day units, a challenge expected to intensify due to a projected 77% increase in global cancer burden by 2050. [7] Capacity constraints in oncology care, exacerbated by a shortage of healthcare professionals, including nurses, are among the most pressing challenges in delivering timely and effective cancer care. [8,9]

In response, a fixed-dose combination subcutaneous (SC) formulation of PT (SC-PT) approved by the European Medicines Agency in 2021, offers an injection time of 5–8 min. [10] While both formulations provide equal clinical outcomes, the SC option presents several potential advantages, including increased healthcare system capacity, patient satisfaction, and resource efficiency. [11] PT-SC could reduce patient time in daycare units and lessen nursing workload, alleviating staffing pressures, increasing patient throughput, and reducing treatment societal costs. [[12], [13], [14]]

Although SC mAb administration reduces patient chair time and healthcare professional (HCP) involvement compared to IV [13,15,16], real-world data on the specific time and cost differences between PT-SC and PT-IV administration remains limited. Given the growing strain on oncology care delivery, understanding these differences is crucial for optimizing healthcare delivery and resource allocation. Therefore, this study aims to identify, quantify, and compare the resource use and costs associated with the administration of PT-SC and PT-IV from a societal perspective.

## 2 Material and methods

### 2.1 Study design

The EASE-SC study was a cross-sectional, observational study employing a bottom-up microcosting approach to quantify and compare the resource use and costs of drug preparation and administration PT-SC versus PT-IV from a societal perspective. The study also estimated the nationwide impact of switching all PT-IV patients in the Netherlands to PT-SC.

The study was conducted between September 2023 and June 2024 at Alrijne Hospital (Leiden), where patients received PT-IV as standard care, and Erasmus University Medical Center Cancer Institute (Rotterdam), where patients received either PT-SC or PT-IV. Eligible participants were patients diagnosed with HER2+ breast cancer and receiving a loading or maintenance dose, administered either as monotherapy or in combination with chemotherapy.

### 2.2 Data collection

Drug preparation and administration data of PT-SC and PT-IV were collected through observations using standardized case report forms (CRFs), capturing disposables used and active HCP time.

Drug preparation times were measured with a stopwatch (minutes:seconds) at the drug preparation area of the hospital pharmacy or oncology daycare unit (Erasmus University Medical Center), recording three phases: material collection, preparation, and finishing. Drug administration data were collected at the oncology daycare unit, with a maximum of two treatment cycles per participant to avoid overrepresentation. Patient chair time was measured as the duration from when patients occupied a chair to their discharge. Administration-related activities and definitions are detailed in **Supplementary Appendix** Table 1. Stopwatch data captured nurses' active time on drug administration tasks, including medication double-checks, administration activities, and other patient care tasks (e.g., monitoring vitals, patient interaction, checking infusion pumps).

For each drug administration, the number of vials used (rounded to whole vials) was calculated based on the prescribed dose. Our study distinguished between loading and maintenance doses, with patients receiving a loading dose for the initial administration, followed by maintenance doses for subsequent administrations (see **Supplementary Appendix** Table 2). Moreover, a material analysis was conducted to determine disposable use during to the drug preparation and administration of PT-SC and PT-IV. Disposable quantities were averaged and standardized per administration, assuming consistent usage within each drug formulation group. Societal resource use was estimated using the validated Institute for Medical Technology Assessment (iMTA) Productivity Cost Questionnaire (iPCQ) completed by patients. [17]

### 2.3 Resource valuation

The analysis included two cost categories: I) healthcare costs, comprising drug costs, disposable costs, patient chair time costs, and active HCP time costs; and II) societal costs, covering travel expenses, informal care costs, and productivity losses due to absences from paid and unpaid work. Unit prices are summarized in Supplementary Table 3.

Costs were estimated following the Dutch Costing Manual (2024) [18,19], with prices adjusted to 2024 euros using the general consumer price index from the Dutch Central Bureau of Statistics if applicable. [20] Drug costs were derived from Dutch list prices. [21] Unit costs of disposables were obtained from the hospital's financial administration. The costs of patient chair time were obtained from Zietse et al. (2024). [22] Unit costs of HCPs were based on wage rates from ‘Collective Labor Agreements’ and were adjusted for social security and holiday surcharges. [23,24] Finally, societal costs were based on reference prices for travel, informal care, and productivity losses. [18]

Total costs were derived by multiplying resource use or time spent by their corresponding unit price. To standardize costs from multiple sources, averages were calculated for unit prices (Supplementary Table 3). For societal costs, patient questionnaires provided data on travel expenses, paid work absences within the friction-cost period (≤12 weeks), average weekly working hours, unpaid work absences, and informal caregiving. [25] These outcomes were standardized across the population, assuming independence from administration route, except for missed unpaid work hours, evaluated separately for PT-SC and PT-IV groups.

### 2.4 Analysis

Total time and costs were calculated for PT-SC and PT-IV, distinguishing between mono- and combination therapy (including chemotherapy). For patients receiving PT in combination with chemotherapy, all procedures attributable to chemotherapy, such as infusion and preparation of chemotherapy, administration of premedication, post-infusion scalp cooling, and IV access line installation (applicable only to PT-SC), were systematically excluded from our time measurements (see Supplementary Fig. 1). This ensured that only PT-related activities were captured, allowing valid comparisons across monotherapy and combination therapy settings. Descriptive statistics reported patient chair time, active HCP time, and costs per treatment cycle, with averages and standard deviations (SD). As is common in microcosting studies, no formal power calculation was conducted, as the primary objective was to provide detailed, representative estimates of resource use rather than to test a statistical hypothesis. [26] A one-way deterministic sensitivity analysis was performed to assess the impact of variations in input variables on overall costs (see **Supplementary Appendix** for details).

### 2.5 Nationwide impact analysis

To estimate the nationwide impact of PT-SC on oncology daycare unit capacity and productivity, we assumed a hypothetical scenario where all PT-IV patients in the Netherlands switched to PT-SC. It was assumed that 80% of the 2,250 Dutch pertuzumab/trastuzumab users were treated with PT-IV receiving maintenance doses over an average of 12 cycles per year. [27,28].

The analysis focused on two outcomes: I) annual chair hour savings at oncology daycare units, and II) annual productivity savings for HCPs, measured in full-time equivalents (FTEs). Annual capacity savings were estimated by dividing the total patient chair time saved per year by the average length of stay for a single therapy at the daycare unit, obtained from Erasmus MC. Annual HCP productivity savings were estimated by dividing the total active HCP time saved per year by the average workable hours per year for healthcare personnel, set at 1,543 h. [18]

## 3 Results

### 3.1 Study observations

The study included 28 patients receiving either intravenous (PT-IV, n = 16) or subcutaneous (PT-SC, n = 12) pertuzumab and trastuzumab. We documented 29 observations during drug preparation (19 PT-IV, 10 PT-SC) and 43 during drug administration (24 PT-IV, 19 PT-SC). Treatments included both monotherapy (pertuzumab and trastuzumab only) and combination therapy with chemotherapy. One PT-SC patient was unable to complete the questionnaire due to a language barrier, and one PT-IV observation was excluded due to an adverse event. Detailed observations are presented in Supplementary Fig. 2.

### 3.2 Patient chair time

**Fig. 1:**
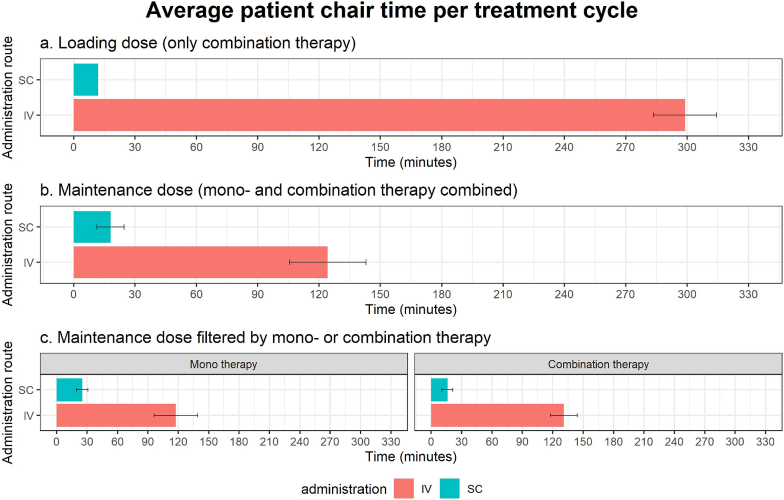
Average patient chair time per treatment cycle observed during drug administration of intravenous (IV) and subcutaneous (SC) pertuzumab/trastuzumab and standard deviation.

PT-SC reduced patient chair time compared to PT-IV (Fig. 1 and Supplementary Table 4). For maintenance doses, the mean chair time decreased by 106.2 min (85.5%), from 124.3 min (PT-IV) to 18.1 min (PT-SC). Loading doses saw a reduction of 287.0 min (96.0%), from 299.0 min (PT-IV) to 12.0 min (PT-SC).

### 3.3 Healthcare professional time

**Fig. 2:**
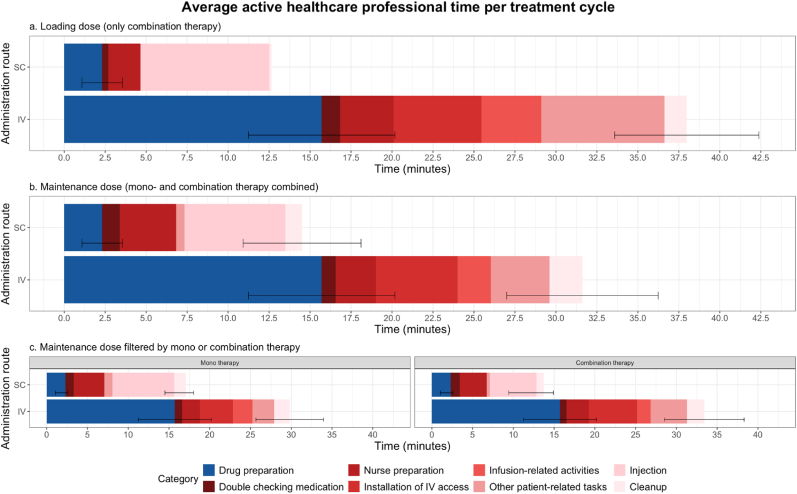
Average active healthcare professional time (HCP) per treatment cycle observed during the drug preparation and administration of intravenous (IV) and subcutaneous (SC) pertuzumab/trastuzumab and standard deviation.

Active HCP time was lower with PT-SC due to shorter preparation and administration durations (Fig. 2 and Supplementary Table 5). Drug preparation time decreased by 13.4 min (85.2%) with PT-SC (2.3 min) versus PT-IV (15.7 min). For maintenance doses, administration time was reduced by 3.7 min (23.3%) with PT-SC (12.2 min) versus PT-IV (15.9 min). Overall, PT-SC reduced total active HCP time by 17.1 min (54.1%) for maintenance doses and 25.3 min (66.7%) for loading doses. In combination therapies, nurse time decreased by 6.3 min (35.6%) with PT-SC, while in monotherapies, PT-SC required slightly more nurse time (0.6 min increase, 4.6%).

### 3.4 Healthcare and societal costs

The administration costs of PT-SC (excluding drug costs) were lower than PT-IV (Table 1). For maintenance doses, total costs were reduced by €171.73 (75.9%), from €226.24 (PT-IV) to €54.52 (PT-SC). Including drug costs, savings were marginal (€7.51, 0.2%). For loading doses, PT-SC reduced total costs by €402.87 (89.8%) excluding drug costs and €238.65 (3.5%) including drug costs.

**Table 1:** Mean costs and standard deviation (SD) associated with drug preparation and administration of intravenous (IV) and subcutaneous (SC) pertuzumab/trastuzumab. HCP: Healthcare professional.

	Maintenance dose					Loading dose				
	SC		IV		Difference (SC - IV)	SC		IV		Difference (SC - IV)
	Average	SD	Average	SD		Average	SD	Average	SD	
**Total drug preparation costs**	**€4.12**	**€0.71**	**€21.82**	**€2.48**	**-€17.70**	**€4.12**	**€0.71**	**€21.82**	**€2.48**	**-€17.70**
Active HCP time	€1.31	€0.71	€8.78	€2.48	-€7.46	€1.31	€0.71	€8.78	€2.48	-€7.46
Disposables	€2.81	**-**	€13.04	–	-€10.23	€2.81	–	€13.04	–	-€10.23
**Total drug administration costs**	**€27.28**	**€7.15**	**€148.82**	**€19.12**	**-€121.54**	**€19.83**	**n/a**	**€329.38**	**€15.86**	**-€309.55**
Patient chair time	€18.18	€6.67	€125.10	€18.83	-€106.92	€12.08	–	€301.05	€15.53	-€288.97
Active nurse time	€8.83	€2.60	€11.51	€3.34	-€2.69	€7.47	–	€16.12	€3.19	-€8.65
Disposables	€0.27		€12.21		-€11.93	€0.27	–	€12.21		-€11.93
IV access line related (e.g., infusion line, IV cannula, flush syringe, etc.)	€-	**-**	€7.87		-€7.87	€-		€7.87	–	-€7.87
Sodium chloride IV solution bag	€-		€0.46		-€0.46	€-	–	€0.46	–	-€0.46
Syringes and needles	€0.12		€3.28		-€3.16	€0.12	–	€3.28	–	-€3.16
Gauzes, bandages, plasters	€0.03		€0.10		-€0.07	€0.03	–	€0.10	–	-€0.07
Protective materials (e.g., gloves)	€0.13		€0.51	–	-€0.38	€0.13	–	€0.51	–	-€0.38
**Total healthcare costs (excluding drug costs)**	**€31.40**	**€7.19**	**€170.64**	**€19.28**	**-€139.24**	**€23.95**	**€0.71**	**€351.20**	**€16.05**	**-€327.25**
**Societal costs**	**€23.12**	**€1.16**	**€55.60**	**€3.27**	**-€32.49**	**€21.66**	**n/a**	**€97.28**	**€2.61**	**-€75.62**
Traveling expenses	€9.78		€9.78		€0.00	€9.78	–	€9.78		€0.00
Informal care	€2.71	€0.99	€18.65	€2.81	-€15.94	€1.80	–	€44.88	€2.32	-€43.08
Productivity losses										–
Paid work	€1.62	€0.59	€11.16	€1.68	-€9.54	€1.08	€0.00	€26.61	€1.21	-€25.54
Unpaid work	€9.01	**-**	€16.01	–	-€7.01	€9.01	–	€16.01	–	-€7.01
**Total costs (excluding drug costs)**	**€54.52**	**€7.28**	**€226.24**	**€19.56**	**-€171.73**	**€45.61**	**€0.71**	**€448.48**	**€16.26**	**-€402.87**
**Drug costs**	**€3,973.05**	**-**	**€3,808.83**	**-**	**€164.22**	**€6,545.45**	**-**	**€6,381.23**	**-**	**€164.22**
**Total costs (including drug costs)**	**€4,027.57**	**€7.28**	**€4,035.07**	**€19.56**	**-€7.51**	**€6,591.06**	**€0.71**	**€6,829.71**	**€16.26**	**-€238.65**

Preparation costs per cycle were €17.70 (81.1%) lower with PT-SC, driven by reductions in HCP time and disposable costs. Administration costs also favored PT-SC, with disposable costs reduced by €11.93 (97.8%). PT-SC resulted in lower societal costs, primarily due to decreased informal care expenses and reduced productivity losses from less time spent at the daycare, with costs €32.49 (58.4%) lower for maintenance doses and €75.62 (77.7%) lower for the loading dose. Traveling expenses were equal for both groups. In contrast, drug costs were higher for PT-SC by €164.22 for maintenance doses (4.3% increase) and loading doses (2.6% increase).

### 3.5 Nationwide impact analysis

The nationwide impact analysis estimates that transitioning all PT-IV patients in the Netherlands to PT-SC could save 38,230 patient chair hours annually, increasing the capacity for an additional 22,059 oncology treatments in daycare units. Additionally, this shift would save 6,157 h of HCP time each year, 4,821 h in drug preparation and 1,336 h in drug administration, which is equivalent to a productivity increase of 4.0 FTEs nationwide.

## 4 Discussion

Our study compared the resource use and costs associated with PT-IV and PT-SC from a societal perspective. The findings demonstrate that transitioning from PT-IV to PT-SC could seriously reduce daycare unit time and nursing workload, alleviate staffing pressures, increase patient throughput, and lower treatment costs.

### 4.1 Comparison with existing literature

While previous studies have indicated that SC administration of mAbs may reduce costs and capacity needs [13,16], few have quantified this for PT-SC. Our study addresses this gap and supports the hypothesis that PT-SC offers substantial efficiency gains. It uniquely combines two mAbs into a single SC formulation, potentially yielding greater savings compared to SC formulations of single agents due to further reductions in preparation and administration times.

Previously, Jackisch et al. (2022) estimated substantial time savings and non-drug cost reductions when switching to PT-SC, based on extrapolations from SC trastuzumab studies and Summary of Product Characteristics (SmPC) information. [12] They reported time savings of up to 250 min for maintenance doses and 532 min for loading doses, with non-drug cost reductions ranging from 73% to 80%. However, our study suggest these estimates are likely overestimated, as they included post-administration observation times up to 360 min for loading doses, as recommended by the SmPC, which are not routinely practiced under our internal safety protocols. In real-world clinical workflows, post-infusion observation is typically shortened or omitted once initial cycles are well tolerated, consistent with local safety protocols and extensive clinical experience. In cases where PT administration is combined with chemotherapy, any observation period is conducted concurrently with the chemotherapy infusion, meaning that it does not result in additional chair time.

Similarly, Munzone et al. (2023) found that switching from PT-IV to PT-SC reduced patient chair time by 87%, active HCP time by 25%, and non-drug costs by €125.95. [29] Their reported savings were slightly lower than ours, likely due to differences in study design and healthcare context. For instance, they included additional activities such as patient registration, blood collection, and oncologist consultations, where time differences between PT-SC and PT-IV were minimal. Their analysis focused on maintenance PT monotherapy and excluded patients on combination therapy. Moreover, healthcare protocols and costs in Italy may differ from our setting. Nevertheless, these findings suggest that similar cost and capacity savings could be achieved in other countries with comparable healthcare structures and treatment protocols, as demonstrated in our study.

### 4.2 Interpretation of findings

The urgency for more efficient administration methods is underscored by capacity constraints, global nursing shortages, and escalating costs of oncologic drugs and care delivery. [8,30,31] As cancer treatments become increasingly complex and expensive, optimizing resource use without compromising patient outcomes is essential to ensure the sustainability of healthcare systems and maintain access to high-quality care. [32]

Our analysis showed that administering PT-SC reduces patient chair time and active HCP time, primarily due to significant reductions in drug preparation time. PT-IV administration involves processes that increase active nurse time, such as installing the IV access line, which can be time-consuming if peripheral venous access is difficult and managing issues like gas bubbles that clog infusion pumps, necessitating nurse intervention. In contrast, PT-SC administration avoids these issues, allowing nurses to focus on the patient's treatment more efficiently. Some patients reported discomfort during PT-SC injections, prompting nurses to slow the injection. Nonetheless, administration remained significantly shorter than with PT-IV.

Our findings also indicate that PT-SC could lead to cost savings compared to PT-IV, primarily due to reductions in overhead and labor costs from decreased patient chair time and active HCP time. While these savings may not directly reduce hospital expenditure, they represent opportunity costs, as freed resources can be reallocated to other treatments. [33] Reducing patient chair time lowers overhead per patient and enables treatment of more patients. [14,29,34,35] Likewise, freeing HCP time improves productivity.

The potential cost savings are highly dependent on drug costs, as shown in the sensitivity analysis. While drug price differences were relatively small, even minor variations can lead to significant absolute cost differences due to the high prices. Our analysis used list prices, but actual purchase prices, negotiated between Dutch hospitals and pharmaceutical companies, are confidential and could hypothetically affect the results. [28]

### 4.3 Strengths and limitations

Our study has several strengths. We used a bottom-up microcosting approach, considered the gold standard for costing studies, as it involves collecting data at the most detailed level. [19,36,37] The study was conducted through direct observations in a real-world clinical setting, enhancing the clinical relevance of our findings. We distinguished between loading and maintenance doses and between monotherapy and combination therapy, allowing time and cost data to be presented at a detailed level. By correcting for time in the combination therapy group, we ensured that only time specifically related to pertuzumab/trastuzumab administration was considered. In addition, we included data from both an academic medical center and a regional general hospital, capturing variation in workflows and costs and thereby increasing the representativeness of our findings.

However, there are limitations to consider. The sample size was modest, particularly for loading doses, with only one observation for PT-SC and four for PT-IV, and four observations in the PT-SC monotherapy group. Therefore, findings regarding loading doses and monotherapy may be influenced by outliers and should be interpreted with caution. Despite this, the variability in observed chair and active nurse times, as reflected by the standard deviations, was small, supporting the robustness of our estimates (maintenance chair time: 18.1 min [SD 6.6] SC vs 124·3 min [SD 18.7] IV; active nurse time: 12.2 min [SD 3.6] SC vs 15.9 min [SD 5.1] IV; Supplementary Tables 4 and 5). Moreover, our findings are consistent with previously published studies on subcutaneous versus intravenous administration of monoclonal antibodies, further supporting their external validity. [15,16,29]

Variations in hospital settings are not expected to substantially impact the incremental total costs (excluding drug costs) to the extent that no cost savings are achieved, as the sensitivity analysis consistently demonstrated negative incremental total costs when varying time and resource use.

### 4.4 Implications for practice and future research

The continual rise in healthcare needs and expenditures poses significant challenges to healthcare systems. [38,39] Efficient allocation of healthcare resources is essential to ensure affordable care and prevent displacement of services.

Our findings indicate that transitioning from PT-IV to PT-SC can save time and potentially reduce costs. However, these savings are highly dependent on drug prices. Hospitals need to assess their individual savings based on their negotiated discounts and reimbursements from payers, as these amounts may not always align with drug list prices. Furthermore, the upcoming expiration of pertuzumab's patent could lead to the introduction of biosimilars into the market. Several biosimilars are currently in development and could substantially reduce PT-IV costs once they enter the market, while PT-SC costs will remain high for the coming years. [40,41] Future research is necessary to evaluate the impact of these new pricing dynamics on overall cost.

While cost savings are important, it is essential to consider other benefits that PT-SC offers, including increased capacity in healthcare settings, improved productivity of HCPs, patient preferences, and reduced material consumption. Moreover, PT-SC holds potential for home-based (self-)administration, which is already standard practice at the Erasmus Medical Center for selected patients. Enabling self-administration could further alleviate capacity constraints, reduce non-drug costs, and potentially increase patient satisfaction by offering greater flexibility and autonomy. [14,42] While home-based self-administration is already common in several therapeutic areas, such as for other subcutaneous biologics and immunoglobulins, oncology has often lagged behind in adopting these models of care. [[42], [43], [44]] Pharmaceutical companies also have an important responsibility to play in facilitating this shift, as current product labels often mandate administration by a healthcare professional, which poses a regulatory and practical barrier to implementing self-administration at scale. [10] Given the potential benefits, further studies are required to investigate the feasibility, patient preference, as well as the time and cost implications, of self-administration at home of PT-SC.

## 5 Conclusions

In conclusion, this study demonstrated that switching from PT-IV to PT-SC can lead to significant reductions in healthcare resource utilization and potential cost savings without compromising patient care. These findings support the adoption of PT-SC as a more efficient administration method for pertuzumab/trastuzumab in HER2-positive breast cancer patients. Future research should focus on exploring the feasibility of home-based self-administration to maximize the benefits of PT-SC.

## CRediT authorship contribution statement

**Michiel Zietse:** Writing – review & editing, Writing – original draft, Methodology, Investigation, Formal analysis, Data curation, Conceptualization. **Jacky Hu:** Writing – review & editing, Writing – original draft, Investigation, Formal analysis, Data curation. **Esther R. van Staveren:** Writing – review & editing, Investigation, Data curation. **Leontine E.A.M.M. Spierings:** Writing – review & editing, Writing – original draft, Supervision, Investigation. **Agnes Jager:** Writing – review & editing, Writing – original draft, Supervision, Investigation, Conceptualization. **Birgit C.P. Koch:** Writing – review & editing, Writing – original draft, Supervision, Investigation, Conceptualization. **Ron H.J. Mathijssen:** Writing – review & editing, Writing – original draft, Supervision, Investigation, Conceptualization. **Roelof W.F. van Leeuwen:** Writing – review & editing, Writing – original draft, Supervision, Investigation, Conceptualization. **Frederick W. Thielen:** Writing – review & editing, Writing – original draft, Supervision, Project administration, Formal analysis, Conceptualization.

## Funding statement

This work was not supported by (external) funding.

## Declaration of competing interest

The authors declare that they have no known competing financial interests or personal relationships that could have appeared to influence the work reported in this paper.
