# Supplementary material for: Capacity and cost benefits of subcutaneous versus intravenous pertuzumab/trastuzumab: The EASE-SC study
Source: Breast. 2025 Sep 10;84:104573. doi: 10.1016/j.breast.2025.104573 (PMC12475515; doi:10.1016/j.breast.2025.104573)
Supplement: Multimedia component 1 [file mmc1.docx]

**Supplementary Appendix**

**Supplementary Table 1:** Overview of administration-related activities and corresponding definitions. PT-IV: Intravenous pertuzumab/trastuzumab; PT-SC: Subcutaneous pertuzumab/trastuzumab.

| **Term** | **Definition** |
| --- | --- |
| Nurse preparation time | Time dedicated to preparatory tasks before administration by the nurse.  PT-IV: Reviewing patient’s file, hanging of infusion bags, collection of drugs and disposables, verification of drug and dose.  PT-IV: Reviewing patient’s file, collection of drugs and disposables, verification of drug and dose. |
| Installing IV access line time* | Time dedicated to the installation of an IV access line. |
| Injection or infusion time | PT-IV: Time between the initiation of the infusion pump to the completion of the infusion pump (including IV line flushing).  PT-SC: Time between the moment the injection needle contacts the skin to the full withdrawal of the injection needle. |
| Cleanup time | Time spent to clean up after administration of pertuzumab/trastuzumab.  PT-IV: Disconnecting IV access line, applying bandages, disposal of materials.  PT-SC: Applying plaster and disposal of materials. |

*Only applicable to PT-IV

**Supplementary Table 2.** Overview of the regulatory approved doses of pertuzumab/trastuzumab for intravenous and subcutaneous administration.

| **Administration route** | **Dose** | **Administration time** | **SmPC Observation Time** |
| --- | --- | --- | --- |
| Intravenous | Pertuzumab 840 mg/trastuzumab 8 mg/kg (loading dose) | 60 min (pertuzumab) + 90 min (trastuzumab) | 60 min (pertuzumab) + 90 min (trastuzumab) |
| Intravenous | Pertuzumab 420 mg/trastuzumab 6 mg/kg (maintenance dose) 3-weekly | 30–60 min (pertuzumab) + 30 min (trastuzumab, if tolerated) | 30–60 min (pertuzumab) + 30 min (trastuzumab, if tolerated) |
| Subcutaneous | Phesgo^©^ (pertuzumab/trastuzumab) 1200 mg/600 mg (loading dose) | 8 minutes | 30 minutes |
| Subcutaneous | Phesgo^©^ (pertuzumab/trastuzumab) 600 mg/600 mg (maintenance dose) 3-weekly | 5 minutes | 15 minutes |

**Supplementary Table 3:** Unit costs of parameters used in the analysis (Euro 2024 prices). N/A: Not applicable; PT-IV: Intravenous pertuzumab/trastuzumab; PT-SC: Subcutaneous pertuzumab/trastuzumab.

| **Parameter** | **Unit costs** | |
| --- | --- | --- |
| **Healthcare professionals wage rates (Source: Collective Labor Agreements) (21, 22)** |  | |
| Wage rate of pharmacists per minute | €0.97 | |
| Wage rate of nurses per minute | €0.72 | |
| Wage rate of pharmacy technicians per minute | €0.54 | |
| **Oncology daycare unit (Source: Similar study conducted at the Erasmus Medical Center)** |  | |
| Overhead costs of oncology daycare unit per minute | €1.01 | |
| **Drug costs (Source: List prices) (20)** |  | |
| Pertuzumab 420 mg (Perjeta®) solution for infusion vial | €2,572.40 | |
| Trastuzumab 150 mg (Herzuma®) solution for infusion vial | €380.44 | |
| Pertuzumab/Trastuzumab 600/600 mg (Phesgo®) solution for injection vial | €3,973.05 | |
| Pertuzumab/Trastuzumab 1200/600 mg (Phesgo®) solution for injection vial | €6,545.45 | |
| **Societal costs (Source: Dutch costing manual) (18)** |  | |
| Car per km | €0.26 | |
| Public transportation per km | €0.22 | |
| Taxi | €3.49 + €2.56 per km | |
| Biking/walking | €0,00 | |
| Informal care costs per hour | €19.51 | |
| Productivity costs per hour (paid work) | €41.40 | |
| Productivity costs per hour (unpaid work) | €19.51 | |
|  | **Erasmus Medical Center** | **Alrijne Hospital** |
| **Disposables related to drug preparation and administration (Source: Hospital financial administration)** |  |  |
| Infusion bag sodium chloride 0.9% 250 mL | €0.42 | €0.50 |
| Water for injection 100 mL | €1.69 | €0.50 |
| Preparation spike with 0.2 µm filter | €0.81 | €0.90 |
| Connection spike | €1.40 | €2.09 |
| Syringe 30 mL | €0.39 | N/A |
| Syringe 20 mL | N/A | €0.41 |
| Syringe 10 mL | €0.15 | N/A |
| Large disinfectant wipe | €0.35 | €0.98 |
| Small disinfectant wipe | €0.03 | N/A |
| Preparation mat | €0.91 | €1.95 |
| Combi-stopper | €0.07 | €0.07 |
| Nonsterile gauze (5x5 cm) | €0.0034 | €0.01 |
| Minigrip bag | €0.24 | N/A |
| Sandwich bag | €0.01 | N/A |
| Biohazard bag | N/A | €1.26 |
| Chemical resistant sterile glove pair | €1.70 | €2.05 |
| Sterile glove pair | N/A | €1.13 |
| Non-sterile glove pair | €0.08 | €0.18 |
| Hairnet | €0.14 | €0.10 |
| Shoe cover pair | €0.11 | €0.86 |
| Isolation gown | N/A | €5.00 |
| Mouth mask | N/A | €0.20 |
| Adhesive cannula dressing | €0.74 | €0.30 |
| Catheter system for infusion | €3.57 | €2.98 |
| Secondary line for infusion | €0.84 | €0.84 |
| Transfer system with spike adapter | €3.92 | €5.60 |
| IV administration set | €2.34 | N/A |
| Adhesive bandages | €2.56 | €2.44 |
| Flush syringe sodium chloride 0.9% 10 mL | N/A | €0.31 |
| Needle for subcutaneous administration | €0.14 | €0.09 |
| Plaster | €0.05 | N/A |


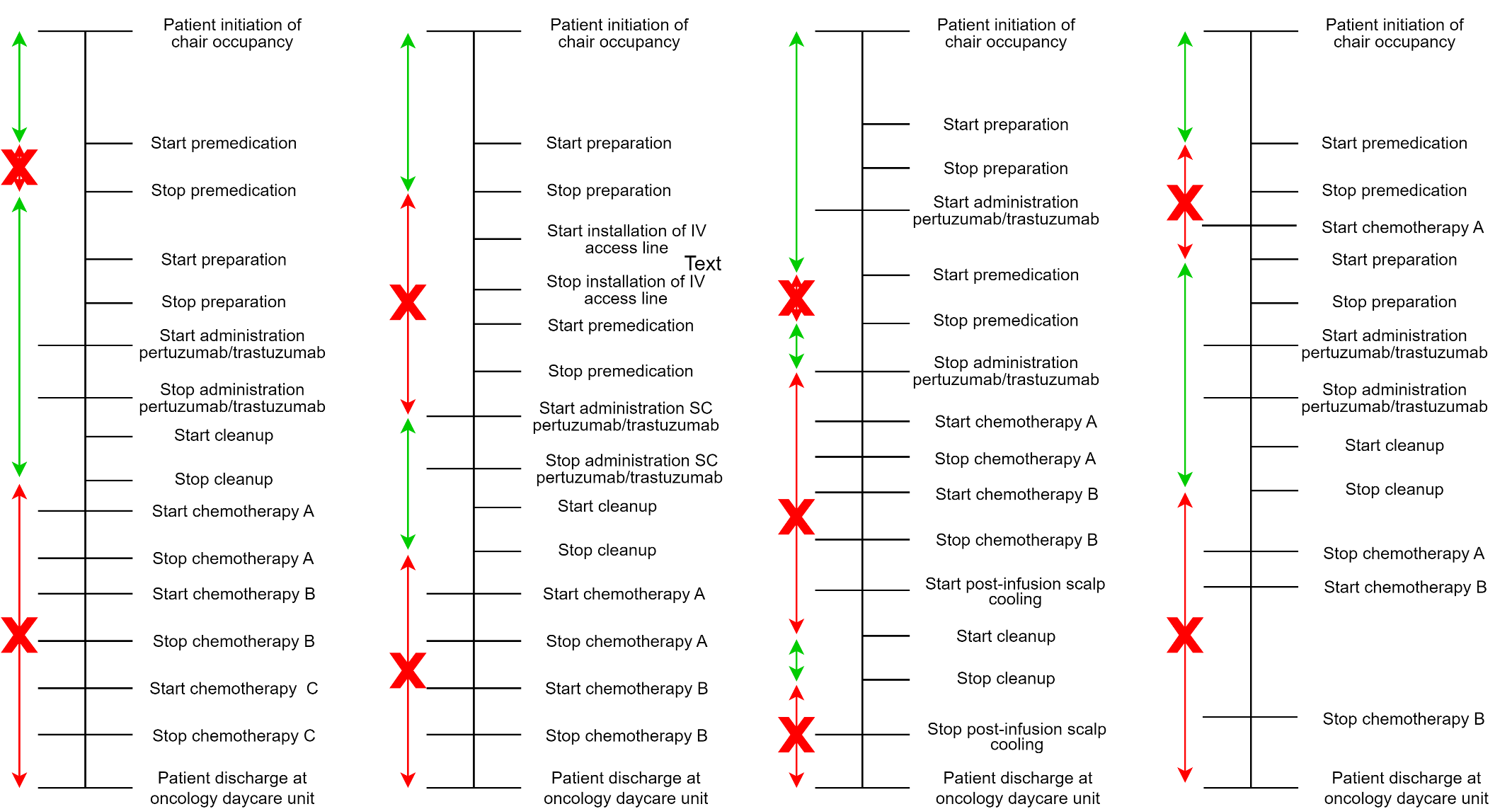


**Supplementary Figure 1:** Illustration of how time corrections were applied during the study. Time corrections for installation of an intravenous (IV) access line time were only applicable for subcutaneous (SC) pertuzumab/trastuzumab. Crosses indicate time unrelated to pertuzumab/trastuzumab, including chemotherapy procedures and IV access installation for SC administration.


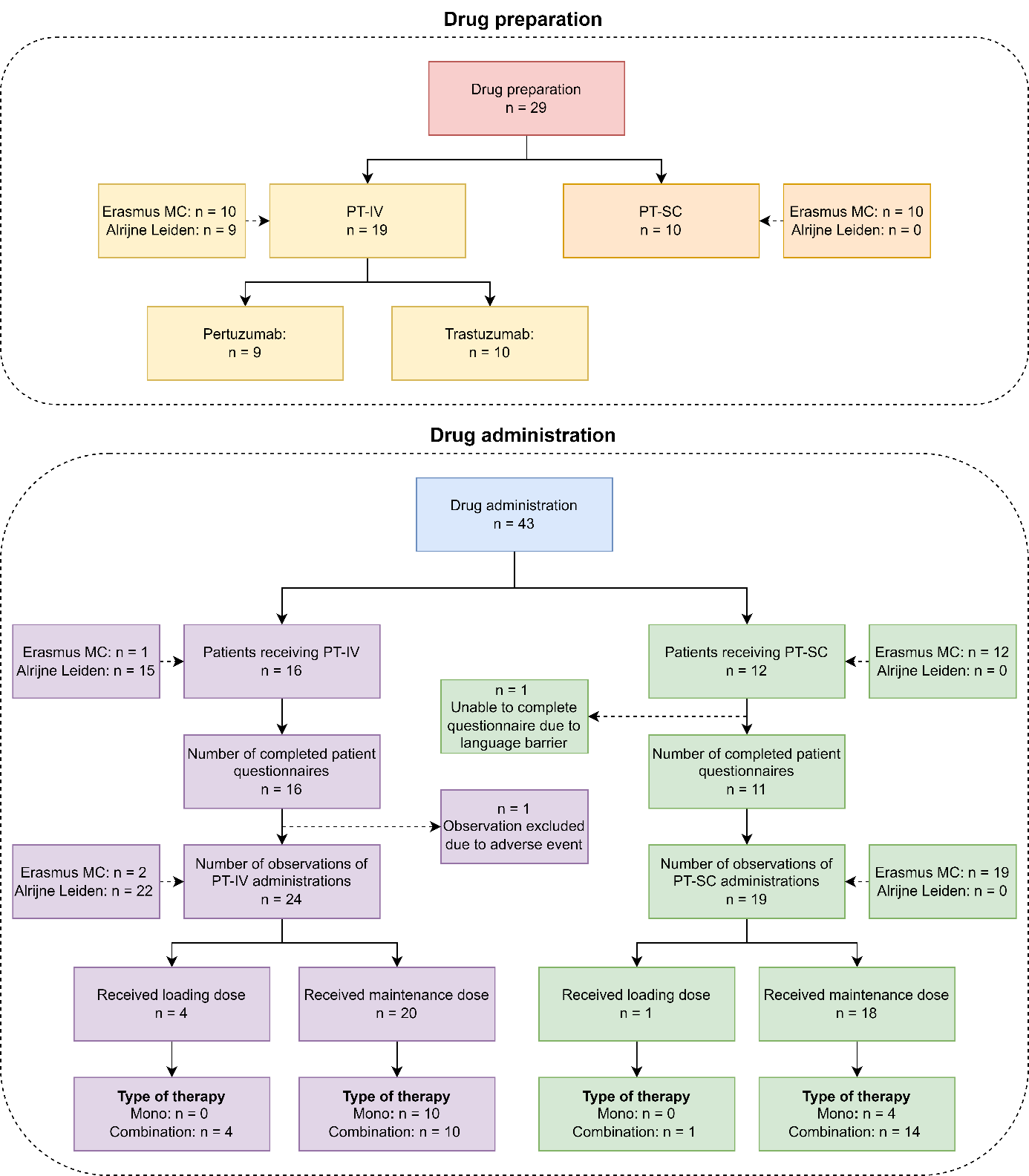


**Supplementary Figure 2:** Breakdown of study observations, including the number of patients, exclusions, locations of activities, type of preparations, type of doses administered, and type of therapy administered. PT-IV: Intravenous pertuzumab/trastuzumab; PT-SC: Subcutaneous pertuzumab/trastuzumab.

**Supplementary Table 4.** Average patient chair time (minutes) with standard deviation (SD) by administration route and treatment type.

| **Treatment Type** | **Intravenous (min, SD)** | **Subcutaneous (min, SD)** |
| --- | --- | --- |
| Maintenance dose | 124.3 (18.7) | 18.1 (6.6) |
| Loading dose | 299.0 (15.4) | 12.0 (n/a) |
| Monotherapy | 117.6 (21.5) | 25.3 (5.6) |
| Combination therapy | 130.9 (13.4) | 16.0 (5.4) |

**Supplementary Table 5.** Average active healthcare professional time (in minutes) with standard deviation (SD), for each task by administration route and treatment type. Values are reported as mean (SD), where available. *Active Nurse Time* is the sum of all individual nurse-related activities including preparation, double-checking, IV installation, infusion-related tasks, injection, cleanup, and other patient-related tasks.

| **Task** | **Maintenance dose IV** | **Maintenance dose SC** | **Loading dose IV** | **Loading dose SC** | **Monotherapy IV** | **Monotherapy SC** | **Combination therapy IV** | **Combination therapy SC** |
| --- | --- | --- | --- | --- | --- | --- | --- | --- |
| **Drug Preparation Time** | **15.7 (4.1)** | **2.3 (1.2)** | **15.7 (4.1)** | **2.3 (1.2)** | **15.7 (4.1)** | **2.3 (1.2)** | **15.7 (4.1)** | **2.3 (1.2)** |
| **Active Nurse Time** | **15.9 (4.6)** | **12.2 (3.6)** | **22.3 (4.4)** | **10.3 (n/a)** | **14.1 (4.1)** | **14.7 (2.6)** | **17.7 (4.9)** | **11.4 (3.8)** |
| *Nurse preparation* | 2.46 | 3.43 | 3.27 | 1.95 | 2.18 | 3.74 | 2.74 | 3.34 |
| *Double checking medication* | 0.85 | 1.08 | 1.12 | 0.38 | 0.89 | 1.01 | 0.81 | 1.10 |
| *Installation of IV access* | 4.98 | - | 5.36 | - | 4.04 | - | 5.92 | - |
| *Infusion-related activities* | 2.03 | - | 3.64 | - | 2.41 | - | 1.65 | - |
| *Injection* | - | 6.17 |  | 7.87 |  | 7.59 | - | 5.73 |
| *Cleanup* | 2.01 | 1.02 | 1.37 | 0.12 | 1.90 | 1.41 | 2.12 | 0.90 |
| *Other patient-related tasks* | 3.58 | 0.49 | 7.51 | 2.32 | 2.68 | 0.99 | 4.47 | 0.34 |

**Sensitivity analysis**

To assess the robustness of our findings and identify key factors influencing the incremental costs between intravenous (IV) and subcutaneous (SC) administration of pertuzumab/trastuzumab, we conducted a one-way deterministic sensitivity analysis. Parameters expected to have the largest impact on costs were varied individually while holding other variables constant at their base-case values, typically adjusted by ±10%. The baseline incremental costs were €7.51 in favor of IV administration including drug costs and €171.73 excluding drug costs.

The key parameters included in the incremental total costs per treatment cycle (including drug costs) are the Mg dose of trastuzumab (450 - 600), minutes of patient chair time for IV administration (98.00 - 165.00), minutes of active nurse time during drug administration for IV (2.85 - 41.28), minutes of patient chair time for SC administration (8.00 - 31.00), and minutes of active nurse time during drug administration for SC (4.14 - 29.32). Additional parameters are the costs of informal care for IV (€14.71 - €24.77) and SC (€1.20 - €4.65), costs of productivity losses due to paid work for IV (€8.80 - €14.82) and SC (€0.72 - €2.78), and minutes of active pharmacy technician time during drug preparation for IV (7.57 - 22.91) and SC (0.50 - 5.98). The analysis also includes minutes of active pharmacist time during drug preparation for IV (0.00 - 1.93), wage rates for pharmacy technicians (€0.42 - €0.63 per minute) and pharmacists (€0.69 - €1.93 per minute), and wage rates for nurses (€0.52 - €0.85 per minute). Lastly, the minutes of active nurse time during drug preparation for SC (0.00 - 1.00) and for IV preparation are considered.

The results of the one-way deterministic scenario analysis are presented **in Supplementary Figure 3 and 4**. The incremental total costs (including drug costs) were most sensitive to changes in trastuzumab dose. Increasing the trastuzumab dose to its maximum input value (600 mg) substantially decreased incremental total costs to -€287.60, meaning that total PT-SC was less expensive than PT-IV. In contrast, lowering the dose to its minimum input value (450 mg) increased the incremental total costs to a positive value of €91.53 in favor of PT-IV. Other parameters to which total incremental costs were sensitive to change included patient chair time and active nurse time. Specifically, an inverse relationship was observed between varying parameters in PT-SC and PT-IV. Changing patient chair time and active nurse time for PT-SC to their maximum input values increased total incremental costs, while changing to their minimum input values decreased total incremental costs. The opposite was found for PT-IV. Furthermore, changes in patient chair time and active nurse time for PT-SC affected the incremental total costs to a lesser extent compared to PT-IV. The effects of other parameters, including informal care costs, costs of productivity losses due to paid work, wage rates of HCPs, and drug preparation time only had a slight impact on total incremental costs. Varying these parameters to their minimum or maximum input values consistently resulted in negative incremental total costs.

**Supplementary Figure 3:** Results of the one-way deterministic analysis for incremental total costs (including drug costs) per treatment cycle between intravenous (IV) and subcutaneous (SC) pertuzumab/trastuzumab. Base case value: -€7.51; Red bars: Maximum input values; Blue bars: Minimum input values.

**Supplementary Figure 4:** Results of the one-way deterministic analysis for incremental total costs (excluding drug costs) per treatment cycle between intravenous (IV) and subcutaneous (SC) administration of pertuzumab/trastuzumab.
Base case value: -€171.73; Red bars: Maximum input values; Blue bars: Minimum input value
